# Carboplatin response in preclinical models for ovarian cancer: comparison of 2D monolayers, spheroids, ex vivo tumors and in vivo models

**DOI:** 10.1038/s41598-021-97434-w

**Published:** 2021-09-14

**Authors:** Melica Nourmoussavi Brodeur, Kayla Simeone, Kim Leclerc-Deslauniers, Hubert Fleury, Euridice Carmona, Diane M. Provencher, Anne-Marie Mes-Masson

**Affiliations:** 1 Centre de Recherche du Centre Hospitalier de l’Université de Montréal (CRCHUM) and Institut du Cancer de Montréal (ICM), CRCHUM, 900 Saint-Denis, Montreal, QC H2X0A9 Canada; 2 Division of Gynecologic Oncology, Université de Montréal, Montreal, QC Canada; 3 Department of Medicine, Université de Montréal, Montreal, QC Canada

**Keywords:** Cancer models, Gynaecological cancer

## Abstract

Epithelial ovarian cancer (EOC) is the most lethal gynecological cancer. Among the key challenges in developing effective therapeutics is the poor translation of preclinical models used in the drug discovery pipeline. This leaves drug attrition rates and costs at an unacceptably high level. Previous work has highlighted the discrepancies in therapeutic response between current in vitro and in vivo models. To address this, we conducted a comparison study to differentiate the carboplatin chemotherapy response across four different model systems including 2D monolayers, 3D spheroids, 3D ex vivo tumors and mouse xenograft models. We used six previously characterized EOC cell lines of varying chemosensitivity and performed viability assays for each model. In vivo results from the mouse model correlated with 2D response in 3/6 cell lines while they correlated with 3D spheroids and the ex vivo model in 4/6 and 5/5 cell lines, respectively. Our results emphasize the variability in therapeutic response across models and demonstrate that the carboplatin response in EOC cell lines cultured in a 3D ex vivo model correlates best with the in vivo response. These results highlight a more feasible, reliable, and cost-effective preclinical model with the highest translational potential for drug screening and prediction studies in EOC.

## Introduction

Epithelial ovarian carcinoma (EOC) is the most lethal gynecological cancer. In 2019, 22,530 women were diagnosed with EOC in the United States and 13,980 died of the disease^1^. Most women are diagnosed at late metastatic stages III-IV, for which only 30.2% will survive 5 years after diagnosis^2^. Although these patients initially respond to first-line treatment (combination of cytoreductive surgery and platinum-based chemotherapy), most patients will eventually recur and develop resistance^3^. Despite the appeal for personalized medicine, no biomarkers have been clinically accepted to accurately predict first-line therapeutic response. To date, carboplatin chemosensitivity remains the main predictor of EOC clinical prognosis^4^.

Significant research efforts have been invested in the discovery of new cancer treatments, with limited focus on the actual experimental models used to test new agents^5^. The current drug discovery pipeline is dependent on 2D cell culture model systems that are devoid of the inherent complexity of their original tumors, which are better captured by in vivo models. In particular, 2D cultures put into question the proper representation of tumor heterogeneity due to cell selective pressures and remain devoid of immune cells and a tumor microenvironment (TME) including the extracellular matrix (ECM), tumor cell-stromal cell interactions, and additional important components^6^. While 2D cell models remain as the primary method of in vitro preclinical testing, attrition rates of anti-cancer drugs continue to be high and EOC survival remains low. To address this, more reliable and practical preclinical models are needed to study drug response and putative biomarkers.

An ideal preclinical model for drug response has optimal physiologic relevance and downstream analysis compatibility, can be translated in a clinically relevant timeframe, and is affordable^7^. To bridge the gap between the shortcomings of 2D models and animal experimentation, there has been a shift to further develop 3D cell culture systems. Overall, 3D spheroids better mimic the structural cell–cell interactions and the chemical nutrient and oxygen gradients^5,8–10^ featured in different cell layers: proliferative, quiescent, and necrotic^5,9–11^. In the setting of EOC, this model is particularly relevant given the physiologic development of ascites (abdominal fluid accumulation) that contain clusters of cells (spheroids)^12^. Additionally, tumor-derived ex vivo models may better mimic the tumor heterogeneity by preserving original TME characteristics, allowing the study of interactions with cancer-associated cells.

**Figure 1:**
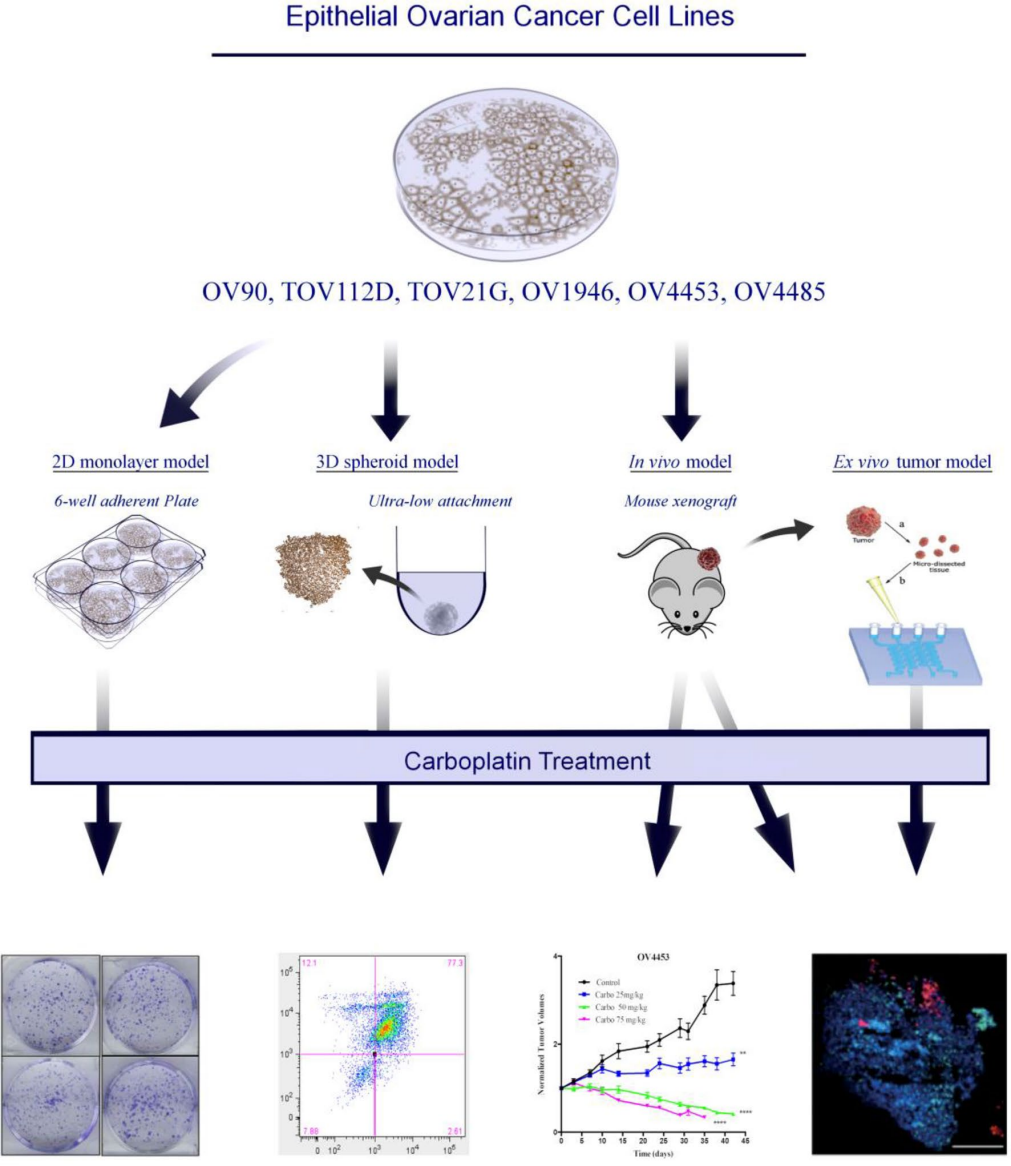
Schematic illustration of study design. Carboplatin chemotherapy response of six EOC cell lines was compared under different model systems: 2D monolayers, 3D spheroids, ex vivo MDTs of tumors, and in vivo xenograft mouse model. Using ultra-low attachment plates, 3D spheroids were treated and analyzed by flow cytometry. Cell lines were injected in immunodeficient mice for xenograft formation and analyzed for tumor volume curves and immunofluorescence. Control xenografts from five cell lines were used for ex vivo tumor generation and placed in microfluidics devices for treatment and analysis by immunofluorescence.

Our group recently showed that response to chemotherapy varies significantly from one in vitro model to another (2D and 3D) for the same cell line and that this variation did not follow the same trend across cell lines^13^. This calls into question the precision of EOC preclinical models, and it is currently unclear whether these in vitro models or an ex vivo model reliably reflects the in vivo gold standard response. To better understand the carboplatin response, we investigated two different in vitro systems (2D monolayers, 3D spheroids) and one ex vivo system (3D micro-dissected tumors^14^) and compared them to the in vivo therapeutic response (xenograft mouse model) (Fig. 1). Using a panel of six EOC cell lines, we show that previously characterized carboplatin response from 2D cultures differs significantly from the in vivo response of the xenograft mouse model. Furthermore, we find that our ex vivo 3D model correlates reliably with the in vivo results. These findings highlight the variability in therapeutic response across model systems and the advantages of a cost-effective 3D ex vivo model for preclinical drug development and testing in EOC.

## Materials and methods

### Cell lines

Six EOC cell lines were selected for their different carboplatin sensitivities (Supplementary Table S1) and their ability to form tumors in immunodeficient mice: OV90 (Cellosaurus ID: CVCL_3768), OV4485 (Cellosaurus ID: CVCL_9T21), OV4453 (Cellosaurus ID: CVCL_9T20), TOV21G (Cellosaurus ID: CVCL_3613), TOV112D (Cellosaurus ID: CVCL_3612) and OV1946 (Cellosaurus ID: CVCL_4375)^13,15–19^. Cell lines originated from patient tumors (TOV) or ascites (OV). OV4485 and OV4453 are *BRCA1* and *BRCA2* mutated cell lines, respectively^16^. Cells were cultured in complete OSE medium (316–030-CL, Wisent Inc, Saint-Bruno, QC, Canada) supplemented with 10% fetal bovine serum (FBS, 088–150, Wisent), 2.5 ug/mL of amphotericin B (450–105-QL, Wisent) and 50 µg/mL of gentamicin (450–135, Wisent). Cells were cultured under conditions previously determined^15–17^. OV90, TOV21G, TOV112D and OV1946 cells were maintained at 21% O_2_ and 5% CO_2_ at 37 °C. OV4485 and OV4453 were kept at 7% O_2_ and 5% CO_2_ at 37 °C. Experiments were carried out with cells between passage 70 to 80 (at ~ 90% confluence). Mycoplasma testing and short tandem repeat (STR) analysis were performed for all cell lines.

### Xenograft mouse model

All animal procedures were performed in accordance with the guidelines for the Care and Use of Laboratory Animals of the CRCHUM as well as the recommendations in the ARRIVE guidelines. This study was approved by the Comité institutionnel de protection des animaux (Animal Ethics Committee, protocol number C18028AMMs). NOD.Cg-Rag1^tm^^1^^Mom^Il2rg^tm1Wjl^/SzJ immunodeficient female mice (007,799, The Jackson Laboratory-JAX, Bar-harbor, Maine, USA)^20,21^ were used to establish xenograft tumors with cell lines. A 200 µL suspension of 1 × 10^6^ cells in 100 µL cold Dulbecco’s PBS (311–425-CL, Wisent) with 100 µL of Matrigel® Matrix (CACB356237, Corning Inc., NY, USA) was injected subcutaneously in the flank of each mouse for the TOV112D, TOV21G and OV90 cells, while 5 × 10^6^ cells were injected for the OV1946, OV4453 and OV4485 cells. Eight mice were used for the control (vehicle) group and for each of the three carboplatin treatment groups per cell line (see section Carboplatin treatment). Treatment was initiated once tumor size was 200 mm^3^ as drug effects can vary if below this value^22^. Mice were between the ages of 11–24 weeks at the start of treatment and given dietary supplementation, DietGel® Recovery and DietGel® Boost (Clear H_2_O, Portland, USA), twice weekly. Tumors were measured with calipers 2–3 times weekly. To alleviate the known negative side effects of carboplatin treatment, anti-nausea medications (1 mg/kg of maropitant and 0.8 mg/kg of ondansetron) were given one hour before the chemotherapy dose and at 24- and 48-h following treatment. Mice were sacrificed at the end of treatment period or if ethical limits were attained through an intraperitoneal injection of euthanyl (pentobarbital sodium) at a dose of 400 mg/kg (concentration of 240 mg/ml). Tumors were collected, measured and were formalin-fixed and paraffin-embedded (FFPE). FFPE tumor blocks were cut into 4 µm sections for histological hematoxylin & eosin (H&E) staining.

### 3D spheroid formation

Rapid, compact and uniform homogenous formation of EOC spheroids was achieved by using 96-well concave-bottom, ultra-low attachment (ULA) microplates (4515/4520, Corning)^8,11,13,16,23^. For all cell lines, 2,000–2,500 cells in 100 µL of complete OSE medium were seeded in each well. Plates were centrifuged at 1,000 rpm for 5 min at room temperature. Spheroids were allowed to form over 48 h in their respective incubation conditions (see Cell lines), generating spheroids of approximately 500 µm in diameter. Spheroids were treated with three carboplatin concentrations (based on optimized IC_50_ dose ranges; see Carboplatin treatment). For each cell line, 20 spheroids were seeded for each carboplatin concentration as well as control groups. Two replicates per condition, containing 10 spheroids for each replicate, were analyzed for each cell line by flow cytometry. In parallel, 10 untreated spheroids were transferred into microfluidic devices at 48 and 96 h for fixation.

### Micro-dissected tissue (MDT) production from cell line xenograft tumors

The micro-dissection procedure was adapted from previously published work^14,24^. Briefly, tumors were sliced into 1 cm thick sections by a scalpel and placed on the McIlwain™ tissue chopper to obtain 350 µm thick slices. Tumor slices were placed in Hank’s balanced salt solution (HBSS, 311–516-CL, Wisent) supplemented with 10% FBS, 2.5 µg/mL of amphotericin B (Wisent) and 50 µg/mL of gentamicin (Wisent). A biopsy punch of 500 µm (PUN0500, Zivic Instruments, Pittsburgh, USA) pierced tumor slices to produce sphere-like MDTs, which were placed in HBSS supplemented with antibiotics without serum until the loading procedure. The loading, trapping and culturing of MDTs were performed as described in our previous work^14^.

### Fixation of MDTs and spheroids within microfluidic devices

MDTs were fixed with 10% formalin (F6050, Produits Chimiques A.C.P. Chemicals Inc, Saint-Leonard, Qc, Canada) after carboplatin treatment and recovery periods, including respective controls. Untreated spheroids were similarly fixed after 48 and 96 h of formation. All specimens were further processed through the previously published paraffin-embedding lithography procedure to create micro-dissected tissue microarray (MDTMA) blocks^14^, which were cut into 4 µm sections for histological H&E staining. Specimen size shrink after this processing technique as previously reported^14^.

### Carboplatin treatment

#### Xenograft mouse model

Based on pilot toxicity studies, carboplatin (Hospira Healthcare Corporation, Saint-Laurent, QC) treatment in the xenograft models was given once weekly by intraperitoneal (IP) injections at either 25, 50, and 75 mg/kg for up to six cycles. The carboplatin vehicle, 0.9% NaCl solution, was used for controls.

#### 3D spheroid model

Optimization studies with spheroids determined the carboplatin treatment range. Thereafter, spheroids were treated by adding 100 µL of three different concentrations of carboplatin (within 0 to 3000 µM final in-well concentration), whereas the control received 100 µL of complete OSE medium. Spheroids were treated for 24 h followed by a 24-h recovery period, based on literature suggesting that 24 h of drug exposure is required to penetrate the spheroid^25–27^. A 24-h recovery was chosen based on published in vitro studies demonstrating the effect of chemotherapy only after its removal^27,28^ and to mimic the physiologic metabolism of the drug. The optimal concentration range was determined for each cell line for minimal growth inhibition and for an effect well below the 50% threshold.

#### *3D *ex vivo* tumor model*

MDTs obtained from untreated xenograft tumors of our cell lines were treated with 6–7 different carboplatin concentrations based on the IC_50_ values from monolayers and 3D spheroids. Two carboplatin regimens were tested for MDTs: a 10-h treatment induction followed by a 14-h recovery, and a 16-h induction followed by a 24-h recovery.

### Clonogenic survival assay

The IC_50_ values for carboplatin for OV4453, OV4485, TOV112D and OV90 were previously determined by clonogenic survival assay^13,16,19^. Carboplatin sensitivity for the OV1946 and TOV21G cell lines was determined in this study using the same clonogenic assay^16^. Briefly, cells were seeded in a 6-well plate at a volume of 1 mL/well and at a density that allowed the formation of individual colonies (1,000 or 1,500 cells/well for TOV21G or OV1946, respectively). Cells were allowed to adhere for 16 h in 5% CO_2_ at 37 °C. Then an additional 1 mL of medium containing carboplatin (final concentrations 0–100 μM) was added in each well and cells were incubated for 24 h. After this period, medium was completely removed and replaced with fresh OSE complete medium. When colonies became visible at 2X magnification, plates were fixed with cold methanol and stained with a solution of 0.5% blue methylene (Sigma–Aldrich Inc., St. Louis, MO) in 50% methanol. Colonies were counted under a stereomicroscope and reported as percent of control. IC_50_ values were determined using Graph Pad Prism 6 (GraphPad Software Inc., San Diego, CA). Each individual experiment was performed in duplicate and repeated three times.

### Flow cytometry analysis of 3D spheroids

After treatment and recovery, 10 spheroids (one replicate) were pooled per condition and dissociated with trypsin–EDTA (0.05%) for 30–45 s to obtain single-cell suspensions. Two replicates were done per experiment. Single cells were labelled using the LIVE-DEAD™ Fixable Aqua Dead Cell Stain Kit (Thermofisher, Massachusetts, USA) stain at 1:100 dilution. After an incubation of 15 min at room temperature, stained cells were analyzed by flow cytometer, LSR-Fortessa (BD Biosciences, Mississauga, ON), using 405 nm excitation, and fluorescence emission was monitored at 525 nm. Data were analyzed using FlowJo (FlowJo LLC, Ashland, USA), identifying dead cells (stained) and live cells (non-stained). Normalized live and dead cell rates were plotted using GraphPad Prism 6 (GraphPad Software Inc.) to generate dose–response inhibition curves with respective IC_50_ values. Each experimental analysis was performed in duplicate and repeated three times.

### Immunofluorescence (IF) and immunohistochemistry (IHC)

FFPE xenograft blocks were cut into 4 µm sections and placed on Fisherbrand superfrost plus microscope slides (Fisherbrand, Ottawa, Ontario). MDTMA blocks (MDT and spheroids) were sliced into 4 µm thick slices and placed on Matsunami TOMO® hydrophilic adhesion slides (VWR, Mont-Royal, QC, Canada). Treatment response in xenograft and MDT experiments was assessed by IF, and viability in untreated spheroids was assessed by IHC. Anti-Ki-67 antibody (cell proliferation) and DAPI (nuclei detection) were used for xenografts and MDTs. Anti-human mitochondria and anti-cytokeratin 8/18 (human epithelial cancer cells) antibodies were additionally used for MDTs. Lastly, antibodies for IHC in spheroid experiments included anti-Ki-67 and anti-cleaved caspase-3 (CC3, apoptosis).

IF/IHC slides were stained using the BenchMark XT automated stainer [Ventana Medical System Inc. (VMSI), Tucson, AZ]. TOMO slides were incubated at 60˚C for 20 min before staining. For IF staining, antigen retrieval was carried out with Cell Conditioning 1 solution (VMSI) for 60 min. Mouse anti-Ki-67 (1:500) antibody (9449, Cell Signaling Technology, Massachusetts, USA), mouse anti-CK8 (1:200) antibody (MA514428, Lab Vision, Sweden), mouse anti-CK18 (1:200) antibody (6259, SantaCruz, California, USA), and mouse anti-human mitochondria (1:2500) antibody (ab92824, Abcam, Cambridge, UK) were automatically dispensed. Slides were incubated at 37 °C for 60 min. Secondary antibodies (dilution 1:250) including Alexa 488 (A11001, Life Technologies, CA, USA) and TRITC (A11030, Life Technologies) were added at room temperature. For IHC staining, antigen retrieval was carried out automatically with Cell Conditioning 1 solution for 30 min (CC3) and 60 min (Ki-67). Rabbit anti-CC3 (1:200) antibody (9661, Cell Signalling Technology) and mouse ant-Ki-67 (1:500) were automatically dispensed followed by horseradish peroxidase secondary antibody. Counterstaining was achieved with hematoxylin and bluing reagent (VMSI). All sections were scanned with an Olympus BX61 microscope using 20 × 0.75 NA objective with a resolution of 0.3225 μm (Bx61vs, Olympus, Toronto, Ontario).

### IF quantification

Stained tumor sections were quantified using VisiomorphDP software version 2020.08 (VisioPharm, Denmark, http://visiopharm.com).

#### Xenograft experiments

IF filters for DAPI and anti-Ki-67 were DAPI and TRITC, respectively. IF quantification of Ki-67 was calculated as a ratio of the total area of Ki-67 positive cells over the detected tissue core area.

#### MDT experiments

IF filters used for DAPI, epithelial cancer cells, and anti-Ki-67 were DAPI, Alexa-488 and TRITC, respectively. Treatment response was quantified as follows: 1) detection of core surface area through DAPI, 2) detection and calculation of total epithelial area within the core through Alexa-488, 3) identification of nuclei of each epithelial cell and calculating total nuclei area through DAPI, and 4) identification of Ki-67 positive nuclei and calculating total positive area for each stain through TRITC.

### Statistical analyses

Values are expressed as the means ± standard error of the mean (SEM) from at least three independent experiments in the case of 3D spheroids. We used eight tumors per condition per cell line for the xenograft model and 15 MDTs per condition per cell line. Comparison between multiple groups (different carboplatin concentrations) was determined by one-way ANOVA comparison test. The IC_50_ of 3D models was calculated by transforming all concentrations into logarithms, normalizing the response, and performing nonlinear regression analysis (dose–response inhibition equation – variable slope). *P* values < 0.05 were considered significant. All statistical analyses were done using GraphPad Prism version 6 (GraphPad Software Inc., http://graphpad.com).

## Results

### Carboplatin sensitivity of 2D EOC cell cultures differs from the in vivo* response*

The selected EOC cell lines have been extensively characterized^13,15–19^ and represent the diverse range of EOC subtypes (dedifferentiated = TOV112D; clear cell = TOV21G; and high-grade serous = OV90, OV1946, OV4453, OV4485) and response to carboplatin treatment (Supplementary Table S1). Based on clonogenic assays, these EOC cell lines have been classified according to pre-determined cut-offs: carboplatin sensitive cell lines in 2D cultures have IC_50_ values below 1 µM (TOV21G and OV4453) and resistant cell lines have IC_50_ values above 10 µM (TOV112D and OV90). Cell lines with IC_50_ values in between are categorized as intermediate (OV1946 and OV4485). This same 2D carboplatin sensitivity criteria have been used previously by others^29^.

**Figure 2:**
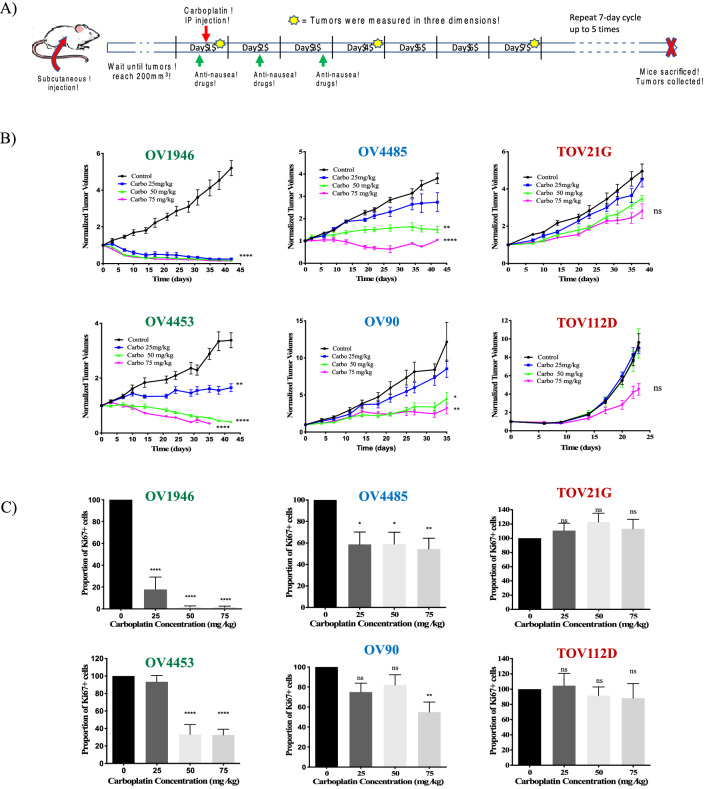
In vivo response of EOC xenografts to carboplatin treatment. **(A)** Timeline for weekly in vivo chemotherapy cycle in our xenograft mouse model. Carboplatin was delivered intraperitoneally (IP). (**B**) Volume measurements of xenograft tumors (N = 8 per condition) throughout carboplatin treatment. (**C**) IF quantification of Ki-67 stain (normalized to control). Sensitive cell lines are indicated in green fonts, intermediate in blue and resistant in red. Data are the mean ± SEM. ns = non-significant. *p < 0.05, **p < 0.01, ***p < 0.001, ****p < 0.0001.

Xenografts were generated from EOC cell lines and treated following the protocol depicted in Fig. 2A. Tumor volumes were recorded throughout carboplatin treatment (Fig. 2B). Chemosensitivity of each cell line was based on inhibition of* in vivo *tumor growth. OV1946 and OV4453 were categorized as sensitive, demonstrating tumor volumes that were significantly lower than the controls at time of sacrifice for all three carboplatin doses (highly responsive). OV90 and OV4485 showed intermediate responses with a significant decrease in tumor volumes at the two highest doses but no response to the lower dose (partially responsive). TOV21G and TOV112D were resistant as they showed no statistical difference at even the highest dose (unresponsive). IF with Ki-67 was quantified from collected xenografts after carboplatin treatment (Fig. 2C) and showed that the response was dose- and cell-line dependent. Results were largely concordant with the tumor volume measurements, confirming chemosensitivity classification. However, treatment response in the xenograft model varied significantly from the 2D culture ranking (Supplementary Table S1); a positive correlation of 2D sensitivity with the *in vivo* response was found in 3/6 EOC cell lines (Table 1).

**Table 1:** Summary of carboplatin sensitivity of EOC cell lines across model systems.

Cell lines	2D monolayers	3D spheroids	3D MDTs	Xenograft	*In vivo* correlation
OV4453	Sensitive	Resistant	Sensitive	Sensitive	2D + 3D MDTs
TOV21G	Sensitive	Resistant	Resistant	Resistant	3D spheroids/MDTs
OV1946	Intermediate	Sensitive	Sensitive	Sensitive	3D spheroids/MDTs
OV4485	Intermediate	Resistant	Intermediate	Intermediate	2D + 3D MDTs
TOV112D	Resistant	Resistant	Resistant	Resistant	2D + 3D spheroids/MDTs
OV90	Resistant	Intermediate	Not performed	Intermediate	3D spheroids

MDTs = micro-dissected tissues.

### Carboplatin response of 3D EOC spheroids improves the correlation with the in vivo response compared to 2D cultures

**Figure 3:**
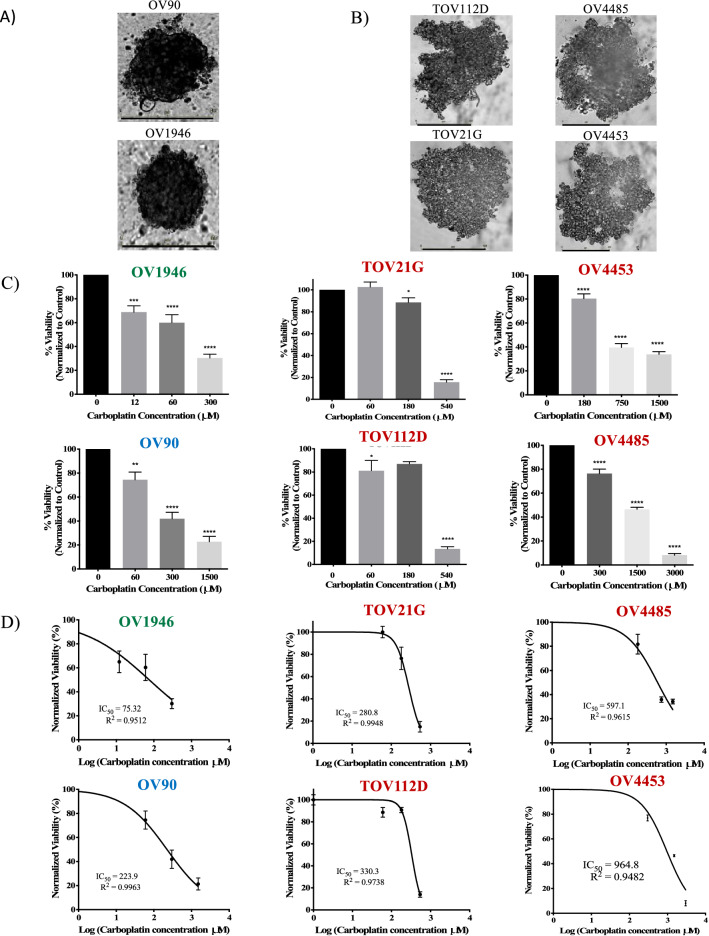
Carboplatin response of EOC 3D spheroids. Representative pictures of EOC cell lines forming at 48 h (**A)** compact spheroids or (**B)** dense aggregates. (**C)** Normalized viability of spheroids after a 24-h carboplatin treatment with a 24-h recovery. (**D)** Dose-inhibition response curves with their corresponding IC_50_. Sensitive cell lines are indicated in green fonts, intermediate in blue and resistant in red. Data are the mean ± SEM of three independent experiments, two replicates per condition. Scale bar on images (**A**, **B**) = 400 µm, *p < 0.05, **p < 0.01, ***p < 0.001, ****p < 0.0001.

All six EOC cell lines formed 3D spheroids in ULA plates. OV90 and OV1946 formed compact spheroids (Fig. 3A), whereas TOV112D, TOV21G, OV4485 and OV4453 formed dense aggregates (Fig. 3B). To demonstrate that cells in the spheroids remained proliferative throughout the experiment, we performed IHC staining to evaluate the level of apoptotic (CC3) and proliferative cells (Ki-67) in spheroids at 48 h (time of spheroid formation) and at 96 h (end of experiment) in the untreated controls (Supplementary Fig. S1). Cells in the spheroids stained strongly for Ki-67 at both time-points with low expression of CC3, demonstrating that they remained proliferative throughout the treatment course.

Flow cytometry was used to evaluate the proportion of viable cells in spheroids after carboplatin treatment (Fig. 3C). IC_50_ values were generated using dose–response inhibition analyses (Fig. 3D). In all cell lines, the 3D spheroid IC_50_ values were significantly higher than that seen in 2D models (Supplementary Table S1). However, the fold change in carboplatin sensitivity between 3 and 2D models varied significantly, depending on the cell line (Supplementary Table S2). The change from 2 to 3D models increased the IC_50_ value by 280-fold for TOV21G, but only sevenfold for OV90, highlighting cell line-dependent changes. Cut-off for resistance to carboplatin treatment was based on response to the physiologic conversion of carboplatin bioavailability (269.4 µM, rounded to 250 µM) in patients (carboplatin dosing of an AUC of 5 corresponding to an average concentration of 300 mg/m^2^, body surface area 1.6 m^2^, blood volume 4.8 L)^30^. Therefore, cell lines with IC_50_ values higher than 250 µM were considered resistant. On the other hand, response to doses below 100 µM were considered sensitive based on previous reports of carboplatin treatment of 3D ovarian cancer models^31,32^. Response between the two cut-offs were considered intermediate. Using these criteria, OV1946 was categorized as sensitive, OV90 as intermediate, and TOV21G, TOV112D, OV4453 and OV4485 as resistant. These results show a positive correlation with the in vivo response for 4/6 EOC cell lines (Table 1).

### The 3D ex vivo tumor model demonstrates a reliable correlation with the in vivo carboplatin response

**Figure 4:**
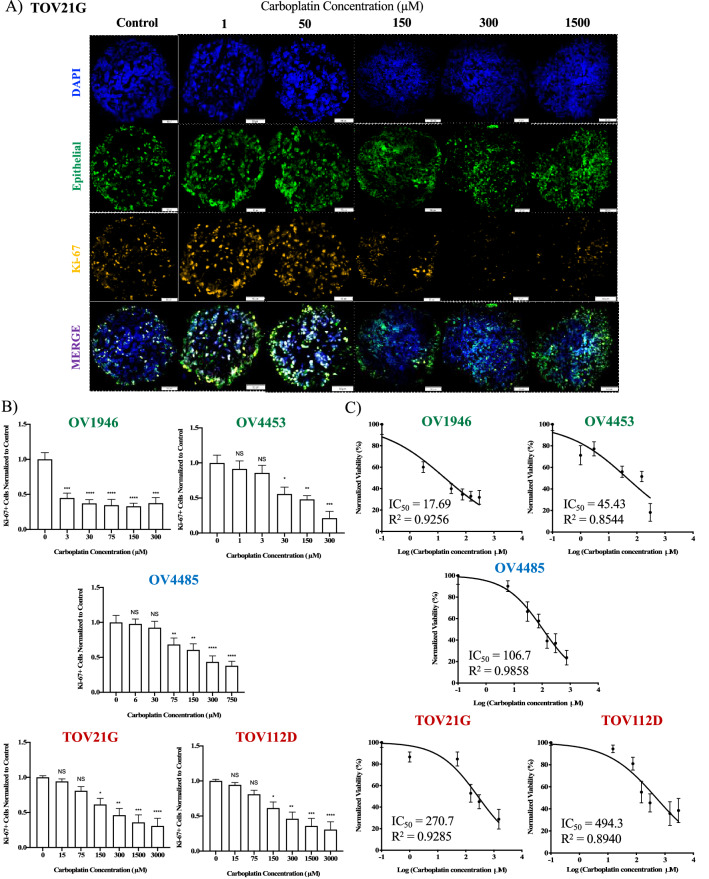
Response of EOC 3D ex vivo tumor model to carboplatin. MDTs derived from several cell line xenograft tumors (OV1946, OV4453, OV4485, TOV21G and TOV112D) were treated with carboplatin at various concentrations (µM) for a 16-h induction followed by a 24-h recovery treatment regimen. (**A)** Representative IF staining for DAPI, mitochondria and cytokeratin 8/18 (human epithelial cancer cells) and Ki-67 of treated TOV21G MDTs. (**B)** Proliferation index, bar graphs are Ki-67 staining normalized to control for each cell line. (**C)** Dose-inhibition curves showing IC_50_ of each cell line. Sensitive cell lines are indicated in green fonts, intermediate in blue and resistant in red. Data are the mean ± SEM. A total of 15 MDTs (technical replicates) were analyzed per condition for each cell line from one xenograft. Scale bar = 50 µm, Magnification = 20x. N.S. = non-significant. *p < 0.05, **p < 0.01, ***p < 0.001, ****p < 0.0001.

Previous studies have shown that MDTs can assess the response to chemotherapeutic drugs in cell line xenograft models^14,24^. Here, we sought to compare the carboplatin sensitivity profiles of cell line xenograft tumor-derived MDTs (Supplementary Fig. S2A) to our ovarian cancer model systems including 3D spheroids and in vivo xenografts using IF analysis (Fig. 4A). The OV90 cell line was excluded for ex vivo analysis due to an insufficient level of cancer cells present in the xenograft tumor precluding the generation of statistically significant results. We first tested two different treatment regimens of carboplatin in our MDTs using OV1946, OV4453 and TOV21G cell lines. This included a 10-h treatment induction followed by a 14-h recovery period (10–14) (Supplementary Figure S2B-D), and a 16-h treatment induction followed by a 24-h recovery period (16–24) (Fig. 4B-C, Supplementary Figure S2B). Both treatment regimens gave similar cell fate responses (proliferation, apoptosis) as well as similar IC_50_ (Supplementary Fig. S2C and S2D, and Fig. 4C). Based on these results, we used the 16–24 treatment regimen to perform the remaining experiments. To compare the chemosensitivity of cell line-based MDTs to 3D spheroids and the in vivo model, we quantified the proliferation capacity of cells after carboplatin treatments (Fig. 4B) and determined IC_50_ values (Fig. 4C). According to our criteria for 3D spheroids, OV1946 and OV4453 were categorized as sensitive (IC_50_ < 100 µM), TOV21G and TOV112D were resistant (IC_50_ > 250 µM), and OV4485 was intermediate. These findings were in complete agreement with the in vivo chemosensitivity results (5/5 EOC cell lines) and provided the best correlation compared to the other two in vitro models (Table 1).

## Discussion

This study highlights the importance of preclinical model selection for drug sensitivity analysis and understanding the variation that exists between experimental models. As most early-phase clinical trial designs rely heavily on preclinical data, it is important to consider these variations when performing drug screening or therapeutic response prediction studies, especially in the era of personalized medicine.

The mainstay of preclinical studies remains cell line-based and the in vivo response from animal models is often used as the gold standard in preclinical testing of novel therapies/combinations. To our knowledge, only one study^33^ using bladder carcinoma cells reported that the 3D spheroids model reflected better the chemoresponse found in their mouse xenograft model, and higher drug resistance was seen with the 3D model compared to 2D cultures. In our study, we compared four translational model systems, including 2D monolayers, 3D spheroids, ex vivo MDTs and in vivo xenografts. Our data suggest better concordance in carboplatin sensitivity between our 3D ex vivo model (MDTs) and in vivo responses. Interestingly, we observed some notable differences between 2D culture and in vivo carboplatin responses. In the case of TOV21G, both its clear cell histology and microsatellite instability^17,34,35^ supports the in vivo response of a platinum-resistant cell line. However, 2D culture experiments have consistently shown this cell line as carboplatin sensitive (Supplementary Table S1 and ^29,36,37^). For OV90 and OV1946, an increased sensitivity to carboplatin is seen in mice. This may be due to their histological high-grade serous subtype of which the majority of patients respond to first-line platinum treatment^3^. Indeed, none of our high-grade serous cell lines showed in vivo resistance to carboplatin, but showed sensitivity in the 2D or 3D spheroid models.

Given that each model has unique features, their relative response to cytotoxic therapy may vary. Immortalized EOC monolayer cultures offer little cell–cell interaction and consist uniquely of a sub-clonal population of epithelial cancer cells. While spheroids also consist of mostly epithelial cancer cells, they offer a 3D structure with inherent cell layers, cell–cell interactions and chemical/nutrient gradients^25,38^. Increasing in model complexity is our ex vivo tumor model of MDTs that not only offers a 3D structure, but also includes mouse-infiltrating stromal cells which may impact the tumor response to a therapeutic agent. In vivo models further increase model complexity by incorporating important elements such as drug metabolism, influence of endogenous hormones and mammalian physiology^39^. In general, our study suggests that the relative carboplatin response of our 3D models was in line with in vivo results. However, two cell lines, OV4453 and OV4485, did not have concordant results as 3D spheroids and demonstrated higher carboplatin resistance in our spheroid model. We suspect that this may be related to their low oxygen culture conditions (7%), which was specific for only these two cell lines. *Hirst *et al*.*^40^ showed that an increase in hypoxia-regulated genes and markers of stemness were present in the core of 3D spheroids but not in monolayered cells and that this induced chemoresistance and phenotypic changes. In addition, these cell lines formed spheroid aggregates that were not compact and had larger spheroid diameters, which has been shown to influence drug resistance^38,41^.

Importantly, our 3D ex vivo model provided a complete concordant correlation with in vivo responses. Ex vivo models are attractive for fundamental and translational research as they can predict patient response to drugs in a clinically relevant timeframe. Important advantages of this model include minimal waste of tissue and culture/drug reagents^24^, control of fluids and constant supply of nutrients^42^, long-term viability^14,43,44^ and the maintenance of MDTs and their TME^14,24^ without need of growth supplements^45,46^. This model further allows testing multiple cycles of cytotoxic therapies as well as studying the effects of cytostatic drugs that require longer incubation periods. This underscores the need to incorporate these models into the drug development pipeline to better evaluate the potential efficacy of new drugs or combinations prior to entering in expensive clinical trial settings.

Although this study has some limitations, such as use of a single drug, choice of flow cytometry for 3D spheroid analyses and limited utility of ex vivo tumors with low epithelial count, the reproducible comparison between model systems while using the same cell lines clearly shows the relevance of using various preclinical models to better characterize response to novel therapies. We are aware that cell lines are often devoid of many elements of the natural TME such as stromal and immune cells, which have been shown to influence response, and may not fully represent the primary tumor heterogeneity. However, the use of cell lines currently remains common practice in most preclinical models. Furthermore, a mouse model may not entirely reflect the human drug response. Hence, ex vivo models derived directly from patient samples would eliminate this bias in the drug development pipeline. Alternatively, similar analyses in comparing these preclinical models could be applied to other drugs in ovarian cancer treatment, such as paclitaxel, poly (ADP-ribose) polymerase (PARP) inhibitors and other drugs currently in preclinical studies such as HIF, WEE1 and TGFß inhibitors. Unfortunately, 3D analysis methods have traditionally relied on 2D culture methods or confocal microscopy analyses, which have their limitations. Thus, it would be interesting to include novel techniques such as light sheet microscopy and tissue clearing as well as IF staining of MDTMAs to allow optimal analysis of tissue without disturbing its natural environment.

Platinum resistance remains an important obstacle in EOC with dismal survival and limited options at advanced stages of disease progression. With the overall high attrition rate of oncologic treatments, more cost-effective predictive cancer models that accurately reflect patient response are needed. With this study, we clearly demonstrate a heterogeneity in therapeutic responses of EOC cell lines when cultured in different systems, which underscores the need to consider multiple factors when selecting a preclinical model for drug discovery and screening studies. This may avoid rejecting potentially effective drugs while eliminating ineffective drugs at the preclinical stage. This could also help reduce the rate of failed clinical trials in which patients experience drug toxicities with minimal efficacy, particularly for rare cancers^23^, which are more difficult to accrue for clinical trials. In the era of personalized medicine, future applications would be to optimize treatment selection based on the individual tumor and patient characteristics rather than a ‘one treatment fits all’ approach. Thus, validation and feasibility studies of newer and more complex models are needed to enhance the current standards.

## Supplementary Information

Supplementary Information.

## Data Availability

The data generated or analyzed during this study are available from the corresponding author upon request.
