# Supplementary material for: Carboplatin response in preclinical models for ovarian cancer: comparison of 2D monolayers, spheroids, ex vivo tumors and in vivo models
Source: Sci Rep. 2021 Sep 14;11:18183. doi: 10.1038/s41598-021-97434-w (PMC8440566; doi:10.1038/s41598-021-97434-w)
Supplement: Supplementary file 1 — Supplementary Information. [file 41598_2021_97434_MOESM1_ESM.docx]

**SUPPLEMENTARY TABLES AND FIGURES**

**Table S1. Characteristics and 2D culture carboplatin response of EOC cell lines**

| **Cell line** | **EOC histology subtype** | **Origin of cell line** | **IC_50_ (µM) (clonogenic assay)** | **Chemosensitivity** | **References** | **Cellosaurus number^a^** |
| --- | --- | --- | --- | --- | --- | --- |
| OV4453 | High-grade serous | ascites | 0.23±0.074 | Sensitive | 16 | CVCL_9T20 |
| TOV21G | Clear cell | tumor | 1.0±0.23^b^ | Sensitive | 17 | CVCL_3613 |
| OV1946 | High-grade serous | ascites | 3.4±0.18^b^ | Intermediate | 15 | CVCL_4375 |
| OV4485 | High-grade serous | ascites | 6.1±0.27 | Intermediate | 16 | CVCL_9T21 |
| TOV112D | Dedifferentiated | tumor | 13.9 | Resistant | 17, 18, 19 | CVCL_3612 |
| OV90 | High-grade serous | ascites | 31.8±5.4 | Resistant | 13, 17 | CVCL_3768 |

^a^ Cellosaurus is an online knowledge resource on cell lines (https://web.expasy.org/cellosaurus/)

^b^ Values obtained are from this present study

**Table S2. Fold change in carboplatin IC_50_ values between 2D and 3D models**

| **Cell Line** | **3D IC_50_/2D IC_50_ (Spheroids)** | **3D IC_50_/2D IC_50_ (MDTs)** |
| --- | --- | --- |
| OV4453 | 659.1/0.23 = 2865.7 | 45.43/0.23 = 197.5 |
| TOV21G | 280.8/1= 280.8 | 270.7/1 = 270.7 |
| OV1946 | 75.32/3.4 = 22.1 | 17.69/3.4 = 5.2 |
| OV4485 | 597.1/6.1 = 97.9 | 106.7/6.1 = 17.5 |
| TOV112D | 330.3/13.4 = 24.7 | 494.3/13.4 = 36.9 |
| OV90 | 223.9/31.8 = 7.0 | - |

**Figure S1.** **3D spheroids remain proliferative throughout 96-hour experiment period.** Representative photographs of **A)** sensitive EOC cell line OV1946 and **B)** resistant EOC cell line TOV112D at spheroid formation (48 hours) and at the end of the experiment (96 hours). Representative staining for H&E (left), Ki-67 (middle) and cleaved caspase-3, CC3, (right). Scale bar = 100 µm.

**Figure S2. Identification of treatment regimen for *ex vivo* tumor model. A.** Schematic representation of MDTs generated from a tumor surgically removed from a cell line xenograft model. **B.** Timeline used for carboplatin treatment of MDTs with a 10-hour incubation followed by 14-hour recovery (10-14 TR) therapeutic regimen. **C.** Cell fate represented in graphs showing percentage of proliferative, quiescent and apoptotic cells within MDTs with or without carboplatin at various concentrations for each treatment regimen for three cell lines (OV1946, OV4453 and TOV21G). **D.** Dose-inhibition curves for 10-14 TR to determine IC_50_. Data are the mean + SEM. A total of 15 MDTs were analyzed per condition for each cell line from one xenograft.
